# Integrated Proteomic and Functional Analyses Reveal the Roles of Organelle-Specific Small Heat Shock Proteins (sHSPs) in Tomato Thermotolerance

**DOI:** 10.3390/plants15111590

**Published:** 2026-05-22

**Authors:** Bolun Xie, Hui Zhou, Huiling Liu, Chenglang Li, Yuhao Song, Yipei Xie, Yanyan Yan, Li Tian

**Affiliations:** 1 Collaborative Innovation Center for Efficient and Green Production of Agriculture in Mountainous Areas of Zhejiang Province, College of Horticulture Science, Zhejiang A&F University, Hangzhou 311300, China; 2023614021057@stu.zafu.edu.cn (B.X.); 2023614022082@stu.zafu.edu.cn (H.Z.); 202320150203@stu.zafu.edu.cn (H.L.); 2024614021023@stu.zafu.edu.cn (C.L.); 2024614021048@stu.zafu.edu.cn (Y.S.); 202424060105@stu.zafu.edu.cn (Y.X.); 2 Key Laboratory of Quality and Safety Control for Subtropical Fruit and Vegetable, Ministry of Agriculture and Rural Affairs, Zhejiang A&F University, Hangzhou 311300, China

**Keywords:** tomato, high temperature, proteome, SlsHSP1, SlHSP17.4

## Abstract

Global warming-induced extreme heatwaves present a severe threat to global tomato yield and production stability. To elucidate the molecular regulatory mechanisms underlying heat stress tolerance in tomato (*Solanum lycopersicum*), this study utilized label-free quantitative proteomics to profile alterations in protein abundance in tomato leaves in response to heat stress. A total of 294 differentially expressed proteins (DEPs) were identified, with function enrichment in the systematic activation of core stress-responsive biological processes, including the mitogen-activated protein kinase (MAPK) signaling cascade, the endoplasmic reticulum protein processing, and glutathione metabolism. Among them, heat shock protein (HSP) family members exhibited the most significant changes, particularly two small heat shock proteins (sHSPs), designated as SlsHSP1 and SlHSP17.4. Functional validation showed that silencing either *SlsHSP1* or *SlHSP17.4* drastically impaired heat tolerance in tomato plants. Specifically, silenced lines displayed excessive reactive oxygen species (ROS) accumulation and reduced antioxidant enzyme activities, with SlsHSP1-silenced plants showing more severe heat-induced phenotypic damage. Subcellular localization assays further demonstrated SlsHSP1 was located in the ER and SlHSP17.4 in the nucleus. Collectively, this study unravels multiple heat defense regulatory networks in tomato, in which organelle-specific sHSPs like SlsHSP1 and SlHSP17.4 synergistically maintain protein homeostasis and cellular redox balance, conferring broad-spectrum stress resistance in plants under high-temperature stress.

## 1. Introduction

Global warming has driven the frequent occurrence of extreme high-temperature events, and heat stress is thus widely recognized as one of the primary abiotic stresses constraining global agricultural production [1]. High-temperature stress irreversibly damages plant growth and development by disrupting key physiological processes such as photosynthesis, membrane fluidity, protein synthesis, enzyme activity, and nutrient uptake [2,3]. Under severe heat stress, the plant cytoskeleton collapses, ultimately triggering catastrophic structural failure of cells [4]. Additionally, heat stress alters the conformation of unstable intracellular macromolecules, inducing protein misfolding and aggregation, and further causing protein toxicity stress [5]. Misfolded proteins and heat-induced metabolic disturbances promote the accumulation of toxic by-products such as reactive oxygen species (ROS) [6]. Accordingly, high-temperature stress impairs multiple plant growth and developmental processes, including seed germination, root morphogenesis, flowering, and fruit set, ultimately leading to drastic reductions in plant reproductive capacity, yield, and survival rate [1,7].

Due to their sessile nature, plants have evolved a suite of adaptive strategies spanning morphological traits, physiological and biochemical pathways, and molecular regulatory networks to mitigate heat-induced damage [8]. Leaves represent one of the most heat-sensitive plant organs; under high-temperature exposure, leaf curling and wrinkling occur to reduce water loss, while severe stress triggers chlorosis, dehydration, and accelerated abscission. In *Arabidopsis thaliana*, the large surface-to-volume ratio of leaves enables intracellular macromolecules (e.g., protein complexes, cell membranes, and nucleic acid polymers) to simultaneously perceive heat signals, theoretically qualifying these molecules as potential heat sensors [9,10]. In the canonical plant heat stress response pathway, heat-induced accumulation of unfolded proteins titrates molecular chaperones away from their inhibitory complexes with heat shock factor (HSF) monomers. Liberated HSF monomers subsequently undergo trimerization and phosphorylation, bind to heat shock response elements (HSEs) in the promoters of target genes, and orchestrate the global heat shock response [11].

Upon heat perception and signal transduction, rapid molecular reprogramming occurs, driving alterations in gene transcription and transcript accumulation to synthesize stress-responsive proteins as a core thermotolerance strategy [12]. To counteract heat stress, plants deploy multiple defensive mechanisms, including the induction of mitogen-activated protein kinase (MAPK), maintenance of membrane stability, ROS scavenging, antioxidant production, and chaperone-mediated protein folding and trafficking [13,14,15]. Under heat stress, the tomato *cpk28* (*calcium-dependent protein kinase 28*) mutant exhibits lower ROS-scavenging enzyme activities compared to wild-type (WT) plants [16]. Activation of the enzymatic antioxidant system is essential for suppressing excessive ROS production and protecting plant cells. For example, knockdown of *LpSGR* (*Staygreen*) reduces the activities of superoxide dismutase (SOD, EC 1.15.1.1), catalase (CAT, EC 1.11.1.6), ascorbate peroxidase (APX, EC 1.11.1.11) and glutathione reductase (GR, EC 1.6.4.2) in transgenic perennial ryegrass, thereby compromising heat tolerance [17]. In tomato, SlHsfA1a transcriptionally regulates the expression of *GST8* (*glutathione S-transferase 8*), *MDAR1* (*monodehydroascorbate reductase 1*), and *Cu/Zn SOD*, enhancing antioxidant enzyme activity and alleviating ROS accumulation in anthers under heat stress [18]. Similarly, heat-stressed pTRV-*HSP70* silenced tomato plants display lower enzymatic activities of SOD, APX, CAT, GR and dehydroascorbate reductase (DHAR, EC 1.8.5.1) and their corresponding transcript levels relative to control pTRV plants [13].

Protein misfolding is a hallmark of heat stress, rendering heat shock proteins (HSPs) indispensable for plant thermotolerance [19]. HSPs, with molecular weights ranging from 10 to 200 kDa, function as molecular chaperones to facilitate proper protein folding and prevent irreversible protein aggregation, thereby safeguarding plants against stress damage and preserving cellular integrity and homeostasis [20,21]. Based on molecular weight, HSPs are classified into distinct families, including HSP100, HSP90, HSP70, HSP60, and small heat shock proteins (sHSPs) [22]. sHSPs represent ubiquitous molecular chaperones, further divided into six subfamilies according to subcellular localization and amino acid sequence homology [23,24]. Mitochondrion-targeted sHSPs promote plant development, modulate photosynthetic efficiency and protein homeostasis under stress [25]. Chloroplast-localized sHSPs are critical for chloroplast development, fruit ripening, and stabilization of photosynthetic complexes [26]. Both nuclear- and plastid-encoded sHSPs contribute to the protective maintenance of photosystem II (PSII) under stress [27]. Endoplasmic reticulum (ER)-localized sHSPs exhibit chaperone activity, shielding target proteins from heat-induced denaturation [28], while peroxisomal sHSPs activate peroxidases to regulate plant stress resistance [29].

Tomato (*Solanum lycopersicum* L.), a warm-season crop originating from the Andean region of South America, is highly vulnerable to high-temperature stress during cultivation [30]. Elevated temperatures inhibit tomato growth, flowering, and fruit set, resulting in severe yield and quality losses [31]. Against this backdrop, we performed label-free quantitative proteomic analysis on tomato plants under heat stress, identifying heat-responsive pathways and differential expression of specific sHSPs. Subsequently, we selected two sHSPs that showed significant changes for further functional analysis via virus-induced gene silencing (VIGS). Our results demonstrated that silencing either of sHSPs significantly impairs the basal thermotolerance of tomato plants. Moreover, subcellular localization assays revealed distinct organellar targeting of these two proteins, suggesting potentially divergent functional roles in the plant heat stress regulatory network. This study not only identifies key sHSPs governing tomato thermotolerance but also provides novel insights into the functional diversification of sHSPs in plant heat defense.

## 2. Results

### 2.1. Proteomic

To explore the molecular mechanisms underlying high-temperature stress in tomato, a label-free quantitative proteomic analysis was performed on leaves subjected to 3 h of high-temperature stress and untreated controls. A total of 276,006 peptides were identified, corresponding to 4405 proteins (Supplementary Table S2). The most of identified peptides were distributed within the range of 7–20 amino acids. Pearson’s correlation coefficients among biological replicates exceeded 0.97 (Supplementary Figure S1A), indicating high data quality and reliable protein identification. Comparative analysis between heat-treated and control samples was conducted to identify differentially expressed proteins (DEPs). Using a threshold of *p* < 0.05, a total of 2112 DEPs were identified, of which 1090 were upregulated and 1022 were downregulated (Supplementary Figure S1B), suggesting widespread proteomic reprogramming in response to heat stress. The subcellular localization of DEPs was predicted using Wolf Psort software (https://wolfpsort.hgc.jp/, accessed on 18 March 2025). As shown in Supplementary Figure S1C, DEPs were predominantly localized to chloroplast, cytoplasm, nucleus, extracellular region, plasma membrane, and mitochondria. The distribution of DEPs across distinct subcellular compartments suggests their coordinated involvement in complex biological networks underlying heat stress adaptation, potentially linking signal transduction, gene expression, energy metabolism, and photosynthesis.

**Figure 1:**
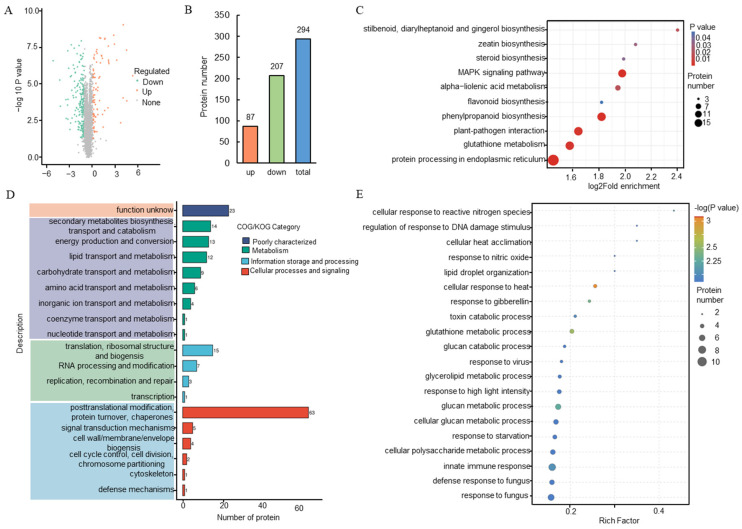
Proteomics changes in tomato in response to high-temperature stress. (**A**) Volcano plot showing alteration in protein abundance induced by heat treatment relative to the control. (**B**) Number of significant DEPs in tomato leaves under heat stress. DEPs were defined as proteins with |fold change| ≥ 1.5 and *p* < 0.05. Orange, green and blue bars represent upregulated proteins, downregulated proteins, and total DEPs. (**C**–**E**) KEGG pathway enrichment analysis (**C**), COG/KOG functional classification (**D**), and biological process enrichment analysis (**E**) of DEPs.

To identify proteins with more pronounced responses to heat stress, a stricter threshold of |fold change| ≥ 1.5 combined with *p* < 0.05 was applied. Under these criteria, 294 DEPs were identified, comprising 87 upregulated and 207 downregulated proteins (Figure 1A,B). KEGG pathway enrichment analysis was performed on the 294 DEPs. The most significantly enriched pathways included the MAPK signaling pathway, phenylpropanoid biosynthesis, and protein processing in the endoplasmic reticulum (Figure 1C). Functional categorization of the DEPs based on eukaryotic orthologous groups (KOG) revealed four major classes, including cellular processes and signaling, information storage and processing, metabolism, and poorly characterized functions. Among these, the category of posttranslational modification, protein turnover, and chaperones was the most highly represented (Figure 1D). These proteins were primarily involved in cellular response to heat, glutathione metabolic process, glucan metabolic process, and innate immune response (Figure 1E).

### 2.2. HSPs Play Crucial Roles in Tomato Thermotolerance

**Figure 2:**
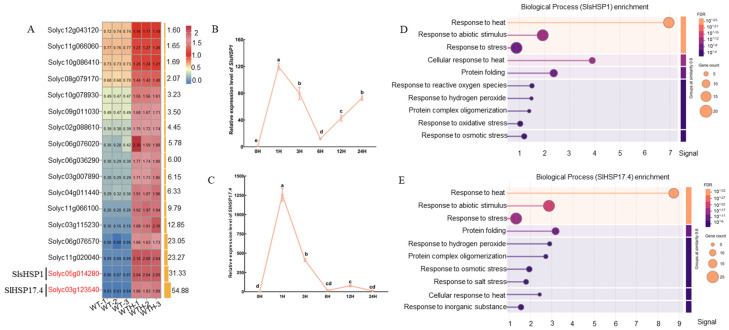
HSPs respond to high-temperatures and participate in diverse biological processes. (**A**) Heatmap of HSPs identified from proteomic data with |fold change| ≥ 1.5 and *p* < 0.05 under heat stress. Detailed information is provided in Supplementary Table S2. The loci Solyc05g014280 and Solyc03g123540 (marked in red) were designated SlsHSP1 and SlHSP17.4, respectively. (**B**,**C**) Relative expression levels of *SlsHSP1* (**B**) and *SlHSP17.4* (**C**) at 0, 1, 3, 6, 12, and 24 h under heat treatment. Biological process enrichment analysis of proteins interacting with SlsHSP1 (**D**) and SlHSP17.4 (**E**). Different letters above bars indicate significant differences at *p* < 0.05 according to Duncan’s multiple range test.

Among the 294 DEPs, 17 HSPs were significantly upregulated under high-temperature stress (Figure 2A, Supplementary Table S3). Two small heat shock proteins (sHSPs), Solyc05g014280 (designated as SlsHSP1) and Solyc03g123540 (designated as SlHSP17.4), exhibited the most pronounced changes. After 3 h of heat stress, SlsHSP1 was upregulated by 31.33-fold and SlHSP17.4 by 54.88-fold relative to the control.

The temporal expression patterns of *SlsHSP1* and *SlHSP17.4* were further examined using RT-qPCR at multiple time points after heat treatment. Both genes were significantly induced within 1 h of heat stress, followed by a gradual decline. Notably, *SlsHSP1* expression showed a secondary upregulation starting at 12 h, whereas *SlHSP17.4* expression remained relatively stable after the initial decline (Figure 2B,C).

Protein–protein interaction (PPI) networks were constructed for SlsHSP1 and SlHSP17.4 using the identified DEPs. The SlsHSP1-centered network comprised 31 protein nodes and 119 interaction edges, which were grouped into 10 functional clusters. Enriched biological processes in this network included response to heat, cellular response to stress, protein folding, and response to oxidative stress (Figure 2D, Supplementary Figure S2A). Similarly, the SlHSP17.4-centered network contained 31 protein nodes and 115 interaction edges. In addition to heat-related processes, this network was also enriched in biological processes such as response to osmotic stress, response to salt stress, response to H_2_O_2_, and protein complex oligomerization (Figure 2E, Supplementary Figure S2B).

### 2.3. Silencing SlsHSP1 Reduces the Resistance of Tomatoes to High-Temperatures

**Figure 3:**
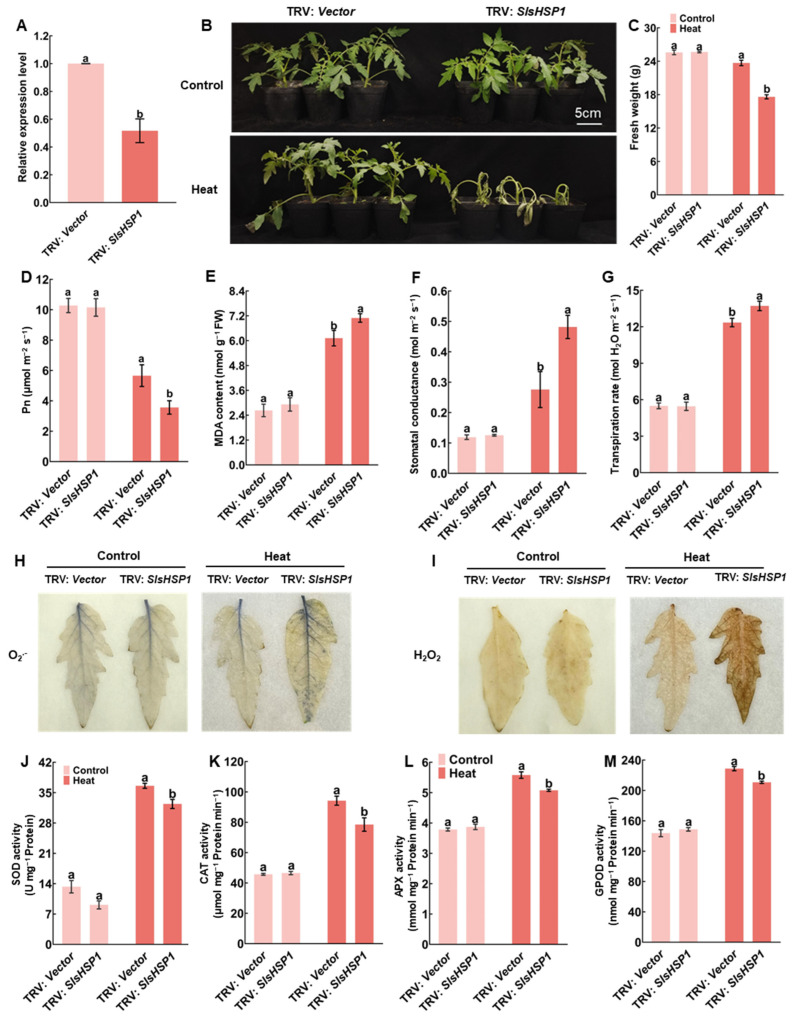
Silencing of *SlsHSP1* compromises thermotolerance in tomato. (**A**) Relative expression level of *SlsHSP1* in TRV:*Vector* and TRV:*SlsHSP1* plants. (**B**) Representative phenotypes of TRV:*Vector* and TRV:*SlsHSP1* plants under heat stress. (**C**–**I**) Physiological indices of fresh weight (**C**), net photosynthetic rate (Pn) (**D**), MDA content (**E**), stomatal conductance (**F**), and transpiration rate (**G**), histochemical staining of O_2_^−^ (**H**) and H_2_O_2_ (**I**) in TRV:*Vector* and TRV:*SlsHSP1* plants under control and heat stress conditions. (**J**–**M**) Activities of SOD (**J**), CAT (**K**), APX (**L**), and GPOD (**M**) in TRV:*Vector* and TRV:*SlHSP1* plants under heat stress. For all panels, at least three biological replicates (individual plants) per treatment group were applied. For fresh biomass, five biological replicates (individual plants) were prepared for each group. For photosynthetic rate, transpiration rate, stomatal conductance and ROS staining, five fully expanded leaves were measured per plant. For antioxidative enzyme activities, three biological replicates were prepared for each group. Different letters above bars indicate significant differences at *p* < 0.05 according to Duncan’s multiple range test.

To investigate the function of *SlsHSP1* in response to heat stress, we silenced this gene using VIGS assay. RT-qPCR analysis confirmed that *SlsHSP1* transcript levels were significantly reduced in *SlsHSP1*-silenced plants (TRV:*SlsHSP1*) compared with empty vector controls (TRV:*Vector*) (Figure 3A). Under normal growth conditions, no significant differences were observed between *SlsHSP1*-silenced plants and TRV:*Vector* controls in terms of phenotype, growth, physiological and injury indicators (Figure 3B–M). Under high-temperature stress, however, *SlsHSP1*-silenced plants exhibited severe wilting and ultimately perished, whereas control plants showed only mild wilting on lower leaves (Figure 3B). The wilting was also reflected by significantly decreased fresh biomass in *SlsHSP1*-silenced plants when compared to TRV:*Vector* control plants (Figure 3C). Photosynthetic capacity in *SlsHSP1*-silenced plants is significantly lower than that in control plants (Figure 3D). MDA content, a marker of lipid peroxidation, was significantly higher in *SlsHSP1*-silenced plants than that in controls (Figure 3E), indicating greater cellular damage. On the other hand, stomatal conductance and transpiration rate were significantly higher in *SlsHSP1*-silenced plants than in controls (Figure 3F,G).

To investigate whether silencing *SlsHSP1* affects ROS homeostasis under heat stress, we examined ROS accumulation using histochemical staining. Under normal conditions, no significant differences in O_2_^−^ and H_2_O_2_ levels were observed among the control plants, *SlsHSP1*-silenced plants, as indicated by NBT and DAB staining, respectively. However, after high-temperature stress, *SlsHSP1* silenced plants exhibited substantially higher ROS accumulation compared with controls (Figure 3H,I). Antioxidant enzyme activity represents an important mechanism for plants to scavenge ROS. Under normal growth conditions, the activities of antioxidant enzymes, including SOD, CAT, APX, and POD, showed no significant differences between the silenced lines and controls. In contrast, under high-temperature stress, the activities of all four enzymes were significantly lower in *SlsHSP1*-silenced plants than in the control plants (Figure 3J–M).

### 2.4. Silencing SlHSP17.4 Impairs Tomato Thermotolerance

**Figure 4:**
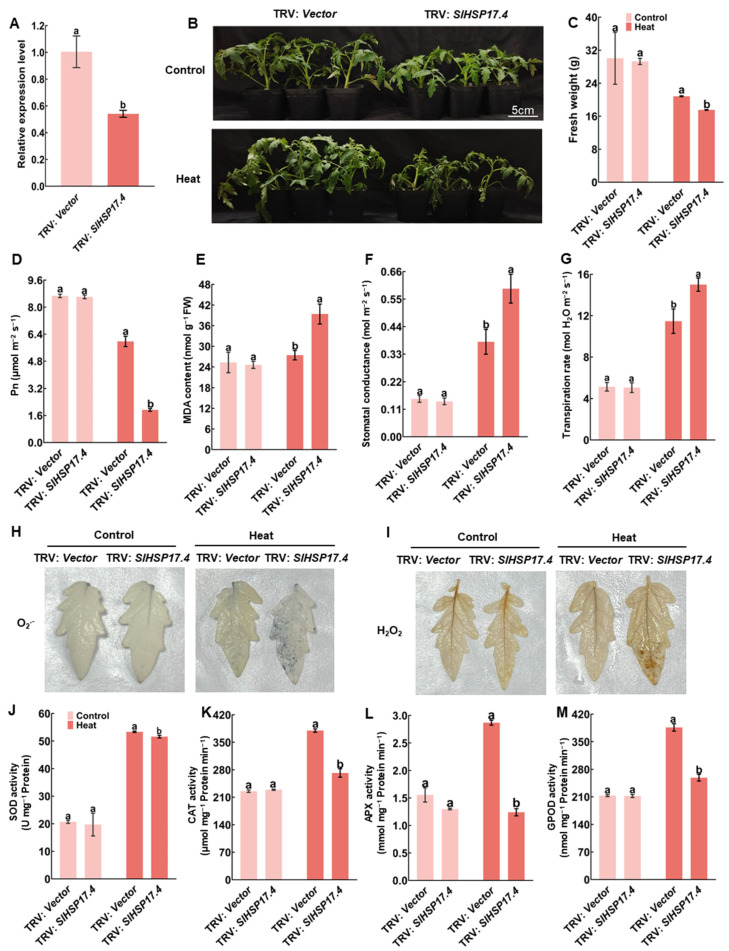
Silencing of *SlsHSP17.4* compromises thermotolerance in tomato. (**A**) Relative expression level of *SlsHSP1* in TRV:*Vector* and TRV:*SlsHSP17.4* plants. (**B**) Representative phenotypes of TRV:*Vector* and TRV:*SlsHSP17.4* plants under heat stress. (**C**–**I**) Physiological indices of fresh weight (**C**), net photosynthetic rate (Pn) (**D**), MDA content (**E**), stomatal conductance (**F**), and transpiration rate (**G**), histochemical staining of O_2_^−^ (**H**) and H_2_O_2_ (**I**) in TRV:*Vector* and TRV:*SlsHSP17.4* plants under control and heat stress conditions. (**J**–**M**) Activities of SOD (**J**), CAT (**K**), APX (**L**), and GPOD (**M**) in TRV:*Vector* and TRV:*SlHSP17.4* plants under heat stress. For all panels, at least three biological replicates (individual plants) per treatment group were applied. For fresh biomass, five biological replicates (individual plants) were prepared for each group. For photosynthetic rate, transpiration rate, stomatal conductance and ROS staining, five fully expanded leaves were measured per plant. For antioxidative enzyme activities, three biological replicates were prepared for each group. Different letters above bars indicate significant differences at *p* < 0.05 according to Duncan’s multiple range test.

Similarly, we silenced *SlHSP17.4* to investigate its function in tomato response to heat stress. RT-qPCR analysis confirmed that SlHSP17.4 transcript levels were significantly reduced in TRV:*SlHSP17.4* plants compared with TRV:*Vector* controls (Figure 4A). After high-temperature stress, *SlHSP17.4*-silenced plants exhibited more severe wilting, lower fresh biomass and higher MDA content compared to the control plants (Figure 4B–D). On the other hand, silencing *SlHSP17.4* reduced photosynthetic rate of tomatoes, increased the stomatal aperture and transpiration rate, and accelerated the water loss process of the plants (Figure 4E–G). Under high-temperature treatment, a higher accumulation of ROS was observed in *SlHSP17.4*-silenced plants (Figure 4H,I), accompanied with lower antioxidant enzyme activities than that in control plants (Figure 4J–M). These results indicate that silencing *SlHSP17.4* impairs the antioxidant capacity of tomato plants under high-temperature stress, thereby resulting in excessive ROS accumulation.

### 2.5. Subcellular Localization of SlsHSP1 and SlHSP17.4

**Figure 5:**
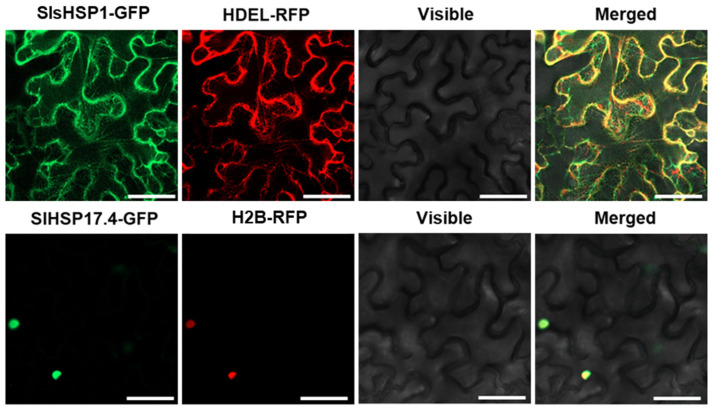
Subcellular location of SlsHSP1 and SlHSP17.4 determined by transient expression in *N. benthamiana* leaves. The full-length coding sequences of *SlsHSP1* and *SlHSP17.4* were fused with the green fluorescent protein (GFP) reporter gene. NbH2B-RFP and HDEL-RFP were used as nuclear and ER markers for co-localization analysis, respectively. Green fluorescence indicates the localization of target proteins, red fluorescence denotes subcellular marker signals, and yellow fluorescence shows their co-localization signals. Scale bars, 50 μm.

To determine the subcellular localization of SlsHSP1 and SlHSP17.4, we generated GFP fusion constructs driven by the CaMV 35S promoter. The resulting constructs were transiently expressed in tobacco leaves, and GFP fluorescence was observed under a laser scanning confocal microscope. Fluorescence colocalization assays revealed that the SlsHSP1-GFP signal overlapped with the ER marker HDEL-RFP, while the SlHSP17.4-GFP signal coincided with the nuclear marker H2B-RFP (Figure 5). These results suggest that SlsHSP1 targets the ER and SlHSP17.4 localizes to the nuclear, implying their divergent functional roles in distinct subcellular compartments.

## 3. Discussion

Climate change-induced high-temperature stress poses a pervasive and escalating threat to agricultural systems worldwide. In plants, high-temperature inflicts multifaceted damage, disrupting photosynthesis, destabilizing cellular membranes, and inducing oxidative stress, ultimately leading to growth inhibition and yield loss [31,32]. At the molecular level, a central aspect of this injury is the widespread denaturation and aggregation of cellular proteins, which compromises essential metabolic functions [33]. While the physiological consequences of heat stress are well documented, plant adaptation and survival depend on a rapid and coordinated reprogramming of the molecular landscape [14]. This complex response, involving signaling cascades, transcriptional activation, and extensive proteome remodeling, cannot be fully deciphered by targeting individual genes or proteins in isolation [34,35]. A comprehensive, systems-level approach is therefore imperative to capture the global dynamics of protein expression and modification under stress. Proteomics serves as a pivotal tool in this endeavor, enabling the unbiased identification and quantification of proteins that directly execute cellular functions.

In this study, we applied comparative proteomic analysis on tomato samples under high-temperature stress to map systemic alterations in protein abundance. After 3 h of heat stress, we identified a set of DEPs distributed across various subcellular compartments (Supplementary Figure S1C). The localization of DEPs in distinct organelles suggests their coordinated participation in a complex biological regulatory network that underpins tomato thermotolerance [36,37]. Our KEGG enrichment analysis revealed that the differentially expressed proteins are significantly enriched in three interconnected pathways, including MAPK signaling, ER protein processing, and glutathione metabolism (Figure 1C–E). MAPK cascades represent the earliest responsive layer. Upon heat stress, membrane fluidity changes and ROS bursts activate specific MAPKKK→MAPKK→MAPK modules [38]. It modulates heat adaptation and survival by fine-tuning ROS homeostasis, hormone signaling (e.g., salicylic acid and abscisic acid), and programmed cell death [38,39]. Upon heat stimulation, the MAPK cascade is rapidly activated and positively regulates the expression of heat shock proteins (HSPs), including small heat shock proteins (sHSPs), by phosphorylating multiple transcription factors such as heat shock factors (Hsfs).

While the observed changes in MAPK-related proteins underscore the activation of upstream signaling cascades essential for initiating transcriptional and adaptive programs, the ultimate thermotolerance phenotype depends on the functional execution of specific biochemical and cellular processes [38]. In line with this, our proteomic profiling revealed significant enrichment of proteins involved in ER protein processing and glutathione metabolism, identifying these as key downstream functional pathways mobilized under thermal challenge (Figure 1D,E). The ER, as the primary site for protein synthesis, folding, and secretion, is highly sensitive to thermal stress, and the accumulation of misfolded proteins within the ER lumen triggers ER stress and the unfolded protein response (UPR) [40]. The observed abundance changes in ER chaperones (e.g., BiP) and protein disulfide isomerases provide direct evidence of the cellular effort to enhance folding capacity, alleviate ER load, and facilitate the degradation or repair of aberrant proteins [41,42]. Glutathione, a central antioxidant metabolite and redox buffer, plays a critical role in scavenging ROS, preventing membrane lipid peroxidation, and protecting protein thiol groups [43]. Accordingly, intact glutathione metabolism is essential for sustaining plant thermotolerance and redox balance under heat stress.

Among the proteins identified within these functional networks, heat shock proteins (HSPs) serve as critical effectors that directly execute protein quality control and cytoprotective functions. Notably, SlsHSP1 and SlHSP17.4 emerged as highly responsive candidates, ranking among the top differentially expressed proteins in our dataset (Supplementary Table S3). Their prominence is not merely a matter of fold change magnitude; rather, their functional positions within the ER-associated folding machinery and redox-protective network make them plausible key determinants of thermotolerance. Specifically, as molecular chaperones, they are well-positioned to alleviate ER stress by facilitating proper protein folding and to mitigate oxidative damage by stabilizing redox-sensitive client proteins. Therefore, beyond the systemic pathway activation described above, we focused on these two HSPs as representative executors of the downstream protective response against high-temperature stress.

To investigate this hypothesis, we employed VIGS to knockdown *SlsHSP1* and *SlHSP17.4* expression, respectively. The increased ROS accumulation and reduced antioxidant enzyme activity observed in silenced lines are consistent with impaired stress tolerance; however, these changes are likely indirect consequences of disrupted proteostasis rather than evidence of direct regulation of ROS metabolism by sHSPs (Figure 3 and Figure 4). Accumulating evidence from multiple plant species shows that sHSPs have no intrinsic ROS-scavenging activity [44]. Instead, they maintain ROS homeostasis by modulating the expression of antioxidant enzyme genes or acting as molecular chaperones. sHSPs can transcriptionally regulate antioxidant-related genes., For example, PpHSP20–26 upregulates the transcript levels of *CAT*, *SOD*, *APX*, *GPX*, and *DHAR* [15]. In addition, several antioxidant enzymes such as CAT are heat-labile and require chaperone-mediated protection under stress [45]. sHSPs can bind to these enzymes and shield them from irreversible protein aggregation [46]. A reasonable interpretation is that the impairment of chaperone function destabilizes core metabolic or regulatory proteins, including those involved in the antioxidant system, thereby exacerbating oxidative stress under high temperature. Low concentration of ROS act as crucial signaling molecules that regulate plant growth and defense responses, whereas excessive and uncontrolled ROS accumulation at high levels results in cellular damage. ROS are produced in multiple subcellular compartments under stress conditions, including chloroplasts, mitochondria, peroxisomes, and the apoplasts [47]. Under heat stress, electron leakage from the photosynthetic and respiratory chains induces H_2_O_2_ production in chloroplasts and mitochondria. These organelles thus serve as both the major source of ROS and the primary targets of heat-induced oxidative damage [48]. In *sHSP*-silenced lines, this regulatory homeostasis is disrupted and ROS accumulation exceeds the physiological signaling threshold, ultimately causing oxidative damage instead of activating stress adaptation mechanisms. Both severe heat shock and prolonged mild heating can induce ROS accumulation, often accompanied by increased expression of ROS-scavenging enzymes [49]. In addition to antioxidant enzymes, glutathione, together with proline, soluble sugars, and polyunsaturated fatty acids, constitutes an important component of the antioxidant defense system and contributes to thermotolerance [50]. Glutathione is particularly vital, as it acts both as a direct ROS scavenger and as a key regulator of cellular redox homeostasis. Based on previous review and our experimental results, we propose a regulatory model whereby heat stress induces controlled ROS production in chloroplasts, mitochondria, and peroxisomes. Nuclear-localized SlHSP17.4 protects HSFs and other transcription factors, thereby promoting the expression of ROS-scavenging enzyme genes. ER-localized SlsHSP1 further safeguards ER-resident components of the antioxidant network and suppresses ER stress-triggered ROS overaccumulation. Collectively, the antioxidant system, including glutathione, SOD, CAT, APX, GR, maintains ROS levels within a physiological range suitable for stress signaling.

sHSPs are a class of widely distributed small molecular chaperones whose core function is to bind and stabilize denatured protein substrates under stress conditions, preventing irreversible aggregation and facilitating the repair or removal of damaged proteins [21]. RT-qPCR analysis revealed that the two *sHSPs* identified in this study exhibited distinct response kinetics to high-temperature stress (Figure 2B,C). The sustained upregulation of *SlsHSP1* suggests a broad and fundamental protein protection function throughout the stress period, reflecting a “continuous defense” strategy. In contrast, *SlHSP17.4* expression tended to stabilize after an initial increase, implying a role concentrated in the rapid response phase at the onset of stress, or potentially selective protection of proteins highly sensitive during early stress. In *Arabidopsis*, sHSPs induced during acute heat shock are thought to provide immediate protection against sudden protein denaturation, whereas those steadily upregulated during acclimation contribute to long-term thermotolerance [51]. Consistent with this paradigm, silencing *SlsHSP1* resulted in a more pronounced loss of thermotolerance, supporting its role as a fundamental and persistent protective factor. The absence of *SlsHSP1* likely exposes the plant to a continuous protein folding crisis throughout the stress period. Silencing of *SlHSP17.4* also results in significant phenotypic changes, but it is less sensitive to high temperatures than silencing of *sHSP1*. Based on the distinct subcellular localizations and the known functional diversity of plant sHSPs, we propose that these two sHSPs are likely non-redundant in their primary functions, yet they may act sequentially and coordinately during different phases of heat stress response, with partial functional overlap under specific contexts.

As SlsHSP1 localizes to the ER and SlHSP17.4 to the nucleus, their client proteins are spatially distinct. ER-sHSP protects secretory pathway components and maintains ER homeostasis. Its putative client proteins are likely nascent or misfolded secretory proteins/transmembrane proteins undergoing synthesis or folding within the ER lumen. In plants, ER-localized sHSPs typically chaperone ER-resident proteases, glycosyltransferases, or secreted defense proteins under heat stress [52]. While nuclear sHSP protects transcription factors and RNA-binding proteins from denaturation, its putative clients are likely nuclear transcription factors, splicing factors, or chromatin-associated proteins that are prone to heat-induced aggregation. Nuclear sHSPs in plants have been shown to protect transcription factors or RNA-binding proteins from denaturation [53]. On the established roles of organelle-specific sHSPs, we hypothesize these two sHSPs are likely non-redundant in their primary functions, yet they may act sequentially and coordinately during different phases of heat stress response. However, whether the two proteins function in complete functional redundancy, partial functional overlap, or act sequentially at different stages of heat stress remains to be further investigated, which could be clarified through genetic analyses using single- and double-mutant lines.

This functional specialization is further supported by their partially distinct protein interaction networks, which suggest differences in their respective target proteins [54]. Preliminary analysis of the protein interaction networks revealed differences in the interaction partners of SlsHSP1 and SlHSP17.4 (Figure 2D,E, Supplementary Figure S2), indicating that these two sHSPs may bind to different substrate proteins or regulatory factors, thereby assuming distinct roles within the cellular protection network. Consistent with this, subcellular localization analysis showed that SlsHSP1 and SlHSP17.4 localize to different organelles, the ER and the nucleus, respectively (Figure 5). Heat stress induces misfolding of nascent polypeptides and membrane proteins, triggering ER stress [55]. The ER-localized SlsHSP1 likely functions as a dedicated chaperone within the ER lumen or at the ER membrane, continuously recognizing, binding, and stabilizing misfolded proteins to alleviate ER stress and maintain ER homeostasis. Its sustained expression may reflect the continuous demand for protein quality control in the ER during heat stress. In contrast, the nucleus serves as the central hub for genetic information and transcriptional regulation [9]. At the early stage of heat stress, acute events such as histone conformational changes, disassembly of transcription complexes, and inactivation of key regulatory proteins occur within the nucleus. The nuclear-localized SlHSP17.4 may function as a rapid response unit that protects the genetic and regulatory machinery during the initial phase of stress. Once the global defense program is successfully initiated, this response may subside, consistent with its expression pattern stabilizing at later time points. Future research should apply co-immunoprecipitation, proximity labeling, or yeast two-hybrid assays to identify direct interaction proteins and delineate the chaperone network for each sHSP, which is essential for dissecting their molecular mechanisms. In addition, exploring whether organelle localization is indispensable for their protective function and whether their subcellular localization alters under stress conditions constitutes an important direction for subsequent study.

It is well established that reproductive organs (anthers, pollen, ovules, and developing fruits) are significantly more sensitive to heat stress than vegetative tissues [56]. While our study focused on vegetative tissues, previous reports demonstrate that sHSPs are expressed in tomato reproductive organs including anthers, ovules, and fruits. For example, tomato *HSP17-CII* is induced in anthers under heat stress and may contribute to fruit set [57]; *LimHSP16.45* shows anther-specific expression in lily pollen mother cells and tapetal cells and functions as a molecular chaperone to protect against temperature extremes [58]. Future studies should examine the tissue-specific expression patterns of *SlsHSP1* and *SlHSP17.4* in flowers and fruits at different developmental stages and generate transgenic lines with fruit-specific overexpression or silencing to directly assess their contributions to fruit set, yield, and fruit quality under heat stress conditions.

## 4. Materials and Methods

### 4.1. Plant Materials and Heat Treatment

Tomato (*S. lycopersicum* L. cv. Ailsa Craig) were cultivated in a constant temperature incubator at 25 °C until they germinated. The germinated seeds grown in a plant growth chamber under a photoperiod of 16 h light (314 µmol m^−2^ s^−1^) at 25 °C and 8 h darkness at 18 °C. When the seedling reached the three-leaves stage, they were randomly divided into the control group (under normal condition) and the high-temperature treatment group. The high-temperature treatment regime consisted of 16 h light (314 µmol m^−2^ s^−1^) at 40 °C and 8 h darkness at 35 °C. Three individual plants were pooled to form one biological replicate, with three biological replicates set for each treatment. Collect samples were collected from both the control group and the group subjected to high-temperature treatment for 3 h, immediately frozen in liquid nitrogen, and stored at −80 °C until protein sequencing.

### 4.2. Quantitative Proteomics Analysis

Protein extraction leaf samples were ground into powder in liquid nitrogen. The powder was transferred to a 5 mL centrifuge tube and sonicated three times on ice using a high-intensity ultrasonic processor (Scientz, Ningbo, Zhejiang, China) in lysis buffer containing 1% Triton X-100, 10 mM dithiothreitol, 1% protease inhibitor cocktail, 50 μM PR-619, 3 μM TSA, 50 mM NAM, 2 mM EDTA, and 1% phosphatase inhibitor. An equal volume of saturated phenol solution (pH 8.0) was added, and the mixture was stirred for 5 min. After centrifugation at 5000× *g* and 4 °C for 10 min, the upper phenol phase was transferred to a new centrifuge tube, and four volumes of ammonium sulfate-saturated methanol were added to precipitate proteins. The mixture was incubated at −20 °C for 6 h, followed by centrifugation at 4 °C and 5000× *g* for 10 min. The resulting pellet was washed three times with cold methanol and three times with cold acetone, respectively. The protein pellet was then dissolved in 8 M urea.

LC-MS/MS analysis Proteins were digested with trypsin, and the resulting tryptic peptides were dissolved in solvent A (0.1% formic acid, 2% acetonitrile in water). Peptides were loaded onto a packed reversed-phase analytical column (25 cm length, 75 μm inner diameter) and separated using a nanoElute UHPLC system (Bruker Daltonics, Rudolf-Plank-Str, Ettlingen, Germany) at a constant flow rate of 450 nL/min. The separation was performed with the following gradient of solvent B (0.1% formic acid in acetonitrile): 6% to 24% over 70 min, 24% to 35% over 14 min, 35% to 80% over 3 min, and held at 80% for 3 min.

Database search and protein quantification The resulting MS/MS data were processed using MaxQuant (version 1.6.15.0) with a decoy database to estimate the false discovery rate (FDR), which was set to 1% at both the peptide and protein levels. Label-free quantification (LFQ) intensities were generated by MaxQuant for each protein across samples. Pearson’s correlation analysis was performed to assess the consistency of quantification among biological replicates. Differentially expressed proteins (DEPs) between the heat-treated group and the control group were identified using Student’s *t*-test. Proteins with a |fold change| ≥ 1.5 and a *p*-value < 0.05 were considered significantly differentially expressed.

Bioinformatics analysis Functional annotation of the identified proteins was performed using the UniProtKB database and the tomato genome database. Subcellular localization was predicted using Wolfpsort. Gene Ontology (GO) annotation was retrieved from the UniProt-GOA database (http://www.ebi.ac.uk/GOA/, accessed on 15 December 2024), and protein IDs were mapped to GO terms. Kyoto Encyclopedia of Genes and Genomes (KEGG) pathway annotation was performed using the KAAS online tool, and pathway mapping was conducted with KEGG Mapper. Enrichment analysis of KEGG pathways was performed using a two-tailed Fisher’s exact test to compare DEPs against all identified proteins. Pathways with a corrected *p* value < 0.05 were considered significantly enriched. Protein–protein interaction networks were analyzed using the STRING database (version 11.0).

### 4.3. VIGS-Mediated Transient Silencing of Target Genes in Tomato for Heat Tolerance Test

To generate gene-silencing constructs, target gene fragments were amplified by PCR using specific primers (Supplementary Table S1) and cloned into TRV2:Vector. The constructs, including TRV1 (assisting in viral infection), TRV2:Vector (as negative control), TRV2:SlsHSP1 and TRV2:SlHSP17.4, were transformed into *Agrobacterium tumefaciens* strain GV3101. A mixture of *Agrobacterium tumefaciens* cultures containing TRV1 and the corresponding TRV2 vectors at a ratio of 1:1 (*v*/*v*), was incubated in the dark at 28 °C, 60 rpm for 3 h, and then injected into tomato seedlings when their cotyledons are fully expanded. After the third true leave emerging, seedlings were transferred to the incubators under normal temperature conditions or a high-temperature incubator with a relative humidity of 70%. The high-temperature stress condition was 16 h light at 40 °C followed by 8 h darkness at 35 °C. When tomato seedlings exhibited phenotypic differences under heat stress, their fresh weight biomass was measured as growth indicator. Each individual plant was defined as one biological replicate. Five biological replicates were included in each group to ensure the reliability and reproducibility of the data.

### 4.4. Photosynthetic Parameters and Oxidative Damage Assessment in VIGS-Silenced Plants

The third fully expanded leaf from each plant was used for these measurements. Net photosynthetic rate (Pn), stomatal conductance (gs), and transpiration rate (Tr) were determined using a portable photosynthesis system (LI-6800, LI-COR, Lincoln, NE, USA) between 9:00–11:00 AM. The following chamber conditions were set: photosynthetically active radiation (PAR) = 324 µmol m^−2^ s^−1^, CO_2_ concentration = 400 µmol mol^−1^ (ambient), block temperature = 25 °C, relative humidity = 60%, and flow rate = 500 µmol s^−1^. For each measurement, the leaf was allowed to equilibrate in the chamber until stable CO_2_ and H_2_O readings were achieved (approximately 2–3 min), and five fully expanded leaves were measured per plant, with three biological replicates per treatment. Chlorophyll content was extracted in 80% acetone and determined by measuring absorbance at 663, 646, and 470 nm [59]. Lipid peroxidation was assessed by measuring the malondialdehyde (MDA) content according to the previously reported method [60]. Each individual plant was defined as one biological replicate. Five biological replicates were included in each group to ensure the reliability and reproducibility of the data.

### 4.5. Determination of ROS and Antioxidative Enzyme Activities

The concentration of superoxide anion (O_2_^−^) and hydrogen peroxide (H_2_O_2_) were determined by nitroblue tetrazolium (NBT) and 3,3′-diaminobenzidine (DAB) staining [61]. For O_2_^−^ staining, the tomato leaves were vacuum-infiltrated in NBT solution (0.1 mg mL^−1^ NBT dissolved in 25 mM K-Hepes buffer, pH 7.8), cultivated at 25 °C in the dark for 2 h and washed with anhydrous ethanol. For H_2_O_2_ staining, leaves were harvested and were vacuum-infiltrated in DAB solution (1 mg mL^−1^ DAB in 50 mM Tris-acetate, pH 3.8) and cultivated at 25 °C in the dark for 24 h, after which the leaves were destained with absolute ethanol.

Tomato leaf samples (0.3 g) were ground with 3 mL of ice-cold 50 mM phosphate buffer (pH 7.8) containing 0.2 mM ethylenediaminetetraacetic acid (EDTA), 2 mM ascorbate, and 2% (*w*/*v*) polyvinylpyrrolidone (PVP). The homogenate was centrifuged at 12,000 rpm and 4 °C for 20 min, and the supernatant was used for the determination of antioxidant enzyme activities. SOD activity was determined by measuring its ability to inhibit the photochemical reduction in nitroblue tetrazolium [62]. CAT activity was determined by monitoring the decrease in absorbance at 240 nm due to H_2_O_2_ decomposition [63]. APX activity was measured by the decrease in absorbance at 290 nm as ascorbate was oxidized [64]. Glutathione peroxidase (GPX, EC 1.11.1.9)) activity was assayed as described by Anderson and Davis [65]. Each individual plant was defined as one biological replicate. Five biological replicates were included in each group to ensure the reliability and reproducibility of the data.

### 4.6. RT-qPCR

Total RNA was extracted using RNA isolater Total RNA Extraction Reagent (Vazyme Biotech, Nanjing, China; cat. No. R401-01). First-strand cDNA was synthesized using the 5 × All-in-one qRT SuperMix (Vazyme Biotech, Beijing, China; cat. No. 7E782C3) according to the manufacturer’s instructions. Quantitative PCR was performed using Power SYBR Green PCR Master Mix (Vazyme Biotech, Beijing, China; cat. No. Q312-02-AA) on a LineGene 9600 Plus real-time PCR system (BIOER, Hangzhou, China) following the manufacturer’s instructions. The cycling conditions were as follows: initial denaturation at 95 °C for 30 s, followed by 40 cycles of 95 °C for 10 s and 60 °C for 30 s. The primer sequences used for RT-qPCR are listed in Supplementary Table S1. The *Actin* gene (gene ID: Solyc11g005330) was used as an internal control [60]. Each reaction was performed with at least three technical replicates. Relative expression levels were calculated using the 2^−∆∆Ct^ method [66], with the untreated control samples serving as the calibrator. We have clarified that two individual plants constitute one biological replicate. Three biological replicates were prepared for each group to ensure data reliability and reproducibility.

### 4.7. Subcellular Localization Analysis

The coding sequences of SlsHSP1 and SlHSP17.4 were amplified from tomato genomic DNA using gene-specific primers (Supplementary Table S1) and cloned into the 35S::GFP vector to generate C-terminal GFP fusion constructs. The resulting constructs were introduced into *A. tumefaciens* strain GV3101. *Agrobacterium* cells harboring the fusion constructs were resuspended in infiltration buffer containing 10 mM MES, 10 mM MgCl_2_, and 150 μM acetosyringone (pH 5.4). The suspensions were infiltrated into the leaves of 4-week-old *Nicotiana benthamiana* plants using a needleless syringe. GFP fluorescence was examined 2 days after infiltration in the epidermal cell layer of the lower leaf surface under a laser scanning confocal microscope (ZEISS LSM 900,Carl Zeiss AG, Oberkochen, Germany) with excitation at 488 nm and emission detected at 505–530 nm.

### 4.8. Date Analysis

Statistical analyses were performed using SPSS software (version 17.0, SPSS Inc., Chicago, IL, USA). The experiment was arranged in a randomized complete block design with three biological replicates per treatment. All data are presented as means ± standard error (SE) from at least three biological replicates. Differences among treatments were assessed using one-way analysis of variance (ANOVA), followed by Duncan’s multiple range test for post hoc comparisons. Statistical significance was set at *p* < 0.05 [67].

## 5. Conclusions

In response to high-temperature stress, tomato plants exhibited a coordinated cellular response characterized by significant enrichment of key biological processes, including the MAPK signaling pathway, ER protein processing, and glutathione metabolism. Among the differentially expressed proteins, the small heat shock proteins (sHSPs) SlsHSP1 and SlHSP17.4 were noted as among the most strongly induced factors. Functional assays showed that silencing either of these genes compromised the antioxidant system, as evidenced by excessive ROS accumulation and reduced antioxidant enzyme activities. Additionally, the two sHSPs exhibited distinct temporal expression patterns, and were localized to different subcellular compartments. Collectively, these findings suggest that SlsHSP1 and SlHSP17.4 may contribute to thermotolerance through differentiated regulatory modules. While the precise architecture of their interaction with broader stress response pathways remains to be defined, this study provides new insights into the functional diversification of sHSPs in plant thermotolerance.

## Data Availability

The original contributions presented in the study are included in the article; further inquiries can be directed to the corresponding authors.

## Supplementary Materials

The following supporting information can be downloaded at: https://www.mdpi.com/article/10.3390/plants15111590/s1, Table S1. Specific primers used for constructing the target gene; Table S2. The DEPs after normal treatment and high-temperature treatment; Table S3. The translation levels of the SlHSP proteins in WT and WTH conditions; Figure S1. Analysis of DEPs under high-temperature stress. (A) Pearson correlation coefficients among different samples. (B) Heatmap analysis of DEPs among samples, with a threshold of *p* < 0.05. WT and WTH in (A) and (B) indicate tomato plants under normal and heat stress conditions, respectively. (C) Subcellular localization classification of DEPs. The x-axis represents the predicted subcellular compartments, and the y-axis represents the number of proteins; Figure S2. Protein–protein interaction (PPI) networks of SlsHSP1 (A) and SlHSP17.4 (B). Nodes represent interacting proteins, and edges indicate different interaction evidence.
