# Supplementary material for: Integrated Proteomic and Functional Analyses Reveal the Roles of Organelle-Specific Small Heat Shock Proteins (sHSPs) in Tomato Thermotolerance
Source: Plants (Basel). 2026 May 22;15(11):1590. doi: 10.3390/plants15111590 (PMC13259247; doi:10.3390/plants15111590)
Supplement: Supplementary file 1 [file plants-15-01590-s001.zip › Supplemental data.pdf]

Supplemental data

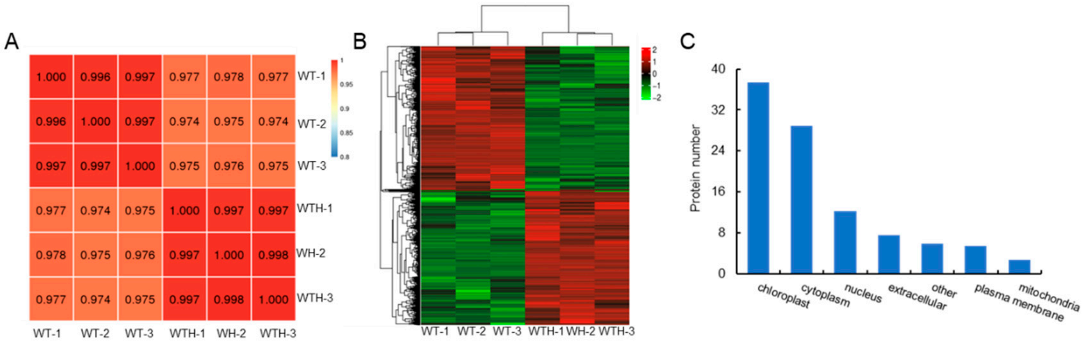

Supplemental Figure S1 Analysis of differentially expressed proteins after high-temperature stress. (A) Pearson correlation coefficients between different samples. (B) Differentially expressed proteins among different samples, and the standards of *p*-value<0.05. (C) Classification of subcellular structure annotations for differentially expressed proteins.

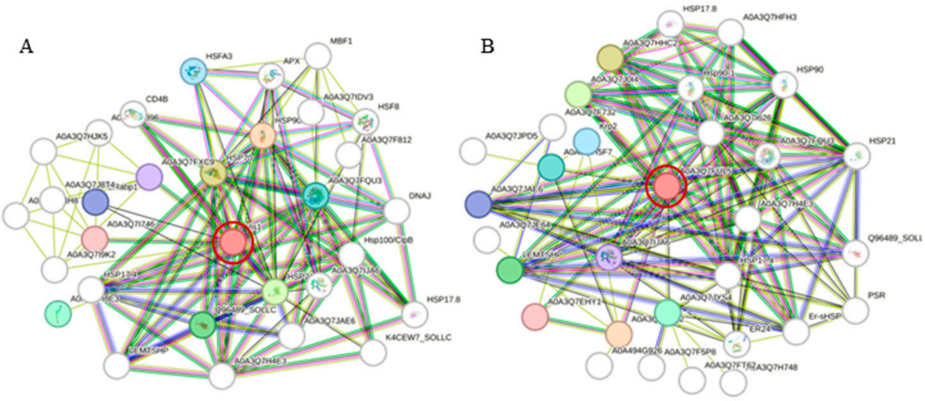

Supplemental Figure S2 Protein-protein interaction (PPI) networks of SlsHSP1 (A) and SlHSP17.4 (B). Nodes represent interacting proteins, and edges indicate different interaction evidence.
